# Structural Mapping of Missense Mutations in the Pex1/Pex6 Complex

**DOI:** 10.3390/ijms20153756

**Published:** 2019-08-01

**Authors:** Anne Schieferdecker, Petra Wendler

**Affiliations:** Institute of Biochemistry and Biology, University of Potsdam, D-14476 Potsdam, Germany

**Keywords:** Zellweger syndrome spectrum disorder (ZSSD), Zellweger, structure, Pex1, Pex6, mutation

## Abstract

Peroxisome biogenesis disorders (PBDs) are nontreatable hereditary diseases with a broad range of severity. Approximately 65% of patients are affected by mutations in the peroxins Pex1 and Pex6. The proteins form the heteromeric Pex1/Pex6 complex, which is important for protein import into peroxisomes. To date, no structural data are available for this AAA+ ATPase complex. However, a wealth of information can be transferred from low-resolution structures of the yeast *sc*Pex1/*sc*Pex6 complex and homologous, well-characterized AAA+ ATPases. We review the abundant records of missense mutations described in PBD patients with the aim to classify and rationalize them by mapping them onto a homology model of the human Pex1/Pex6 complex. Several mutations concern functionally conserved residues that are implied in ATP hydrolysis and substrate processing. Contrary to fold destabilizing mutations, patients suffering from function-impairing mutations may not benefit from stabilizing agents, which have been reported as potential therapeutics for PBD patients.

## 1. Introduction

Peroxisomes are ubiquitous single membrane-bound organelles, which provide pivotal anabolic and catabolic functions. Failure of peroxisomal function manifests in peroxisomal disorders (PDs), which are further classified into single peroxisomal enzyme deficiencies (PEDs) and peroxisomal biogenesis disorders (PBDs). While PEDs concern a single peroxisomal metabolic function, a myriad of peroxisomal functions are scrutinized in PBDs. These autosomal, recessive, nontreatable diseases can be related to any of the 14 human peroxins involved in de novo biogenesis, homeostasis, and proliferation of peroxisomes [1]. Peroxins PEX1, PEX2, PEX5, PEX6, PEX10, PEX12, PEX13, PEX14, PEX26, PEX3, PEX16, and PEX19 are all implicated in the protein import machinery. Variations in these peroxins cause a broad spectrum of clinical presentations of varying severity known as Zellweger syndrome spectrum disorders (ZSSDs). ZSSDs comprise historically distinct diagnoses with decreasing severity: Zellweger syndrome (ZS), neonatal adrenoleukodystrophy (NALD), and infantile Refsum disease (IRD). Recently, Heimler syndrome (HS) was identified as the mildest presentation of a ZSSD [2,3].

ZS affects approximately 1 in 50,000 births in the United States [4]. Most of these cases are attributed to mutations in peroxins Pex1 (60%) and Pex6 (16%) [1,5]. Both proteins form a heteromeric complex that extracts the peroxisomal transporter Pex5 from the transiently formed peroxisomal pore complex. For import into peroxisomes, proteins that contain a peroxisomal targeting sequence are recognized by Pex5 and shuttled to the peroxisomal membrane. Interaction of cargo-loaded Pex5 with Pex14 supposedly facilitates formation of a transient pore. After cargo delivery into the peroxisomal matrix, Pex5 is monoubiquitinated by the peroxisomal RING–finger E3 ligase complex (Pex2, Pex10, Pex12), Pex4, and Pex8. The Pex1/Pex6 complex, which is attached to the peroxisomal membrane via Pex26, recognizes and recovers monoubiquitinated Pex5 from the membrane for new rounds of import. In the yeast *Saccharomyces cerevisiae,* depletion of *sc*Pex1 attenuates the import of matrix proteins [6]. Furthermore, yeast deletion strains of *sc*Pex1 and *sc*Pex6 contain peroxisomal membrane remnants, which contain little to no matrix proteins [7]. These so-called ghost peroxisomes are a common feature found in fibroblasts of ZSSD patients. Their abundance, size, and morphology depend on the nature of mutations carried by the patient [8,9].

Based on investigations into genotype–phenotype correlations, a classification of mutations into class I or II mutations was proposed [10,11,12]. Class II mutations comprise deletions, insertions, frameshifts, truncations, as well as nonsense and splice variants. By tendency, patients carrying a class II/class II genotype show low steady-state mRNA levels and nondetectable protein levels of the respective peroxin [12]. Thus, homozygous class II mutations are associated with a complete loss of peroxisomal function and the severe ZS phenotype that is characterized by a survival of less than 12 months. A prominent example for a homozygous class II mutation is mutation hPex1.I700YfsX42, the second most common Pex1 mutation [13]. Class I mutations comprise missense mutations. The most common Pex1 mutation is hPex1.G843D. Homozygous patients show residual protein levels of Pex1 of approximately 3–20% [12,14]. When fibroblasts derived from homozygous patients are stained with either an anti-catalase or anti-SKL antibody, approximately 20% of fibroblasts display punctuate signals indicating the presence of peroxisomes and residual activity of the import machinery [14].

Generally, the activity of the Pex1/Pex6 complex is indirectly assessed in immunofluorescence experiments by visualization of peroxisomes in patient-derived fibroblasts with anti-catalase or anti-SKL antibodies. Peroxisomes that contain catalase or PTS1 imported matrix proteins appear as punctuate signals. The number of cells that contain peroxisomes, so-called peroxisome-positive cells, compared to fibroblasts from healthy individuals indicate the functionality of the Pex1/Pex6 complex. The impact of individual variations on Pex1/Pex6 functionality can be investigated by expression of the particular variant in cell lines deficient of the respective peroxin. In such complementation assays, peroxisome-positive cells are counted to assess the level of complementation. A temperature-sensitive phenotype was found for the mutation hPex1.G843D: When cultivated at 37 °C, 20% of fibroblasts from patients showed peroxisomes containing catalase and PTS1 imported matrix proteins. This number increased to approximately 90% when fibroblasts were cultivated at 30 °C [14]. It was, therefore, implied that hPex1.G843D structurally destabilized the Pex1/Pex6 complex. Consequently, fold-stabilizing agents like arginine, betaine, and flavonoids were found to enhance peroxisomal import in patients’ fibroblasts [15,16,17]. Patients carrying a homozygous G843D mutation in Pex1 are commonly diagnosed with a mild phenotype, generally IRD, and show a variable postnatal survival of 2 years to above 45 years [12]. Although postnatal survival is a strong indicator of severity, the variability of this parameter is common in patients with class I/class I or class I/class II genotypes. A severity scoring system that quantitatively scores a patient’s phenotype (e.g., ear or facial abnormalities) was not able to correlate the assigned severity score with survival [18]. A study of patients surviving into adulthood that investigated peroxisomal metabolites in fibroblasts of patients’ fibroblasts, plasma, erythrocytes, and urine was also not able to correlate the data on peroxisomal function with the severity of the clinical phenotype [19].

**Figure 1:**
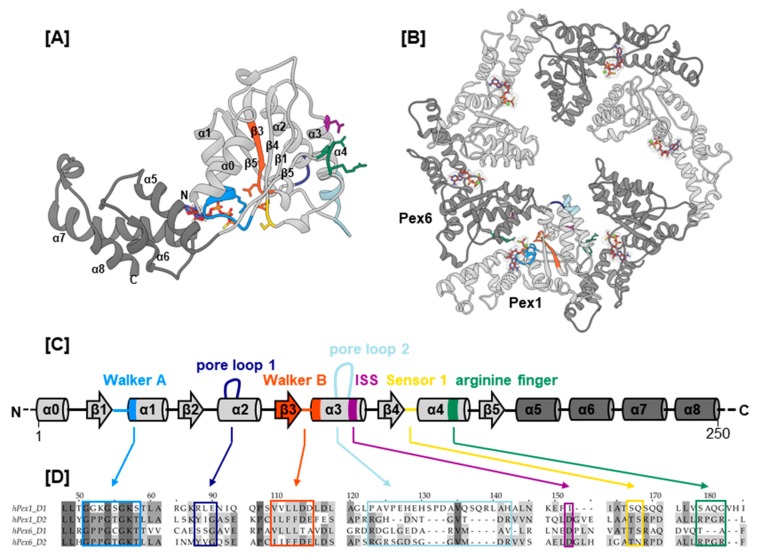
Structure and oligomeric arrangement of the D2 AAA+ ATPase domains. (**A**) Structure of the D2 domain of Pex1. The α/β-Rossmann fold is colored in light grey and the C-terminal α-helical subdomain in dark grey. Secondary structure elements are numbered in the order of appearance from N- to C-terminus, and conserved motifs are color coded. (**B**) Model of the hexameric assembly of the Pex1/Pex6 D2 AAA+ ring bound to ATP. Pex1 and Pex6 domains are colored in light and dark grey, respectively. (**C**) Secondary structure annotation of the Pex1 D2 domain shown in (**A**). (**D**) Sequences of canonical AAA+ elements in all Pex1/Pex6 ATPase domains color coded as shown in (**A**,**C**). The nucleotide is shown as a stick and surface representation in all monomers.

Pex1 and Pex6 belong to the broad superfamily of AAA+ ATPases (ATPases associated with diverse cellular activities; Figure 1). Their closest homologs are *N*-ethylmaleimide sensitive factor (NSF) and p97, which, like Pex1 and Pex6, belong to the classic clade of AAA+ ATPases (Figure S1) [20,21]. AAA+ ATPases are characterized by a structurally conserved ATPase domain, which comprises an α/β-Rossmann fold and a C-terminal α-helical subdomain [22] (Figure 1A–C). The central β-sheet of the α/β-Rossmann fold contains the conserved structural motifs Walker A, which coordinates the γ-phosphate of ATP during hydrolysis, and Walker B, which coordinates the water-activating magnesium ion for hydrolysis. The α/β-Rossmann fold further contains the conserved “second region of homology”, which harbors the sensor 1 motif and two arginine finger residues, as typical for the classic clade of AAA+ domains. Most AAA+ ATPases are only active in a hexameric assembly formed around a central pore (Figure 1B). Upon ATP hydrolysis, the hexamer can undergo significant movements and adopt circular or spiral arrangements [23,24,25,26,27]. For hydrolysis to take effect, the arginine finger of one ATPase domain reaches into the nucleotide binding pocket of the counterclockwise adjacent ATPase domain to interact with the bound nucleotide. Within the AAA+ ring or spiral, subunit contact is maintained by interactions between the α-helical subdomain and the α/β-Rossman fold of the clockwise adjacent ATPase domain. The hexamer is often further stabilized by association of affiliated N- or C-terminal domains. Most AAA+ ATPases remodel the substrate by threading it through the central pore to be unfolded in the process. Electron microscopy (EM) structures of several AAA+ complexes, such as Vps4 (Vacuolar protein sorting-associated protein 4) [26], NSF [28], ClpB [29], YME1 [30], VAT (valosin-containing protein-like ATPase of Thermoplasma acidophilum) [27], and p97 [25], present a hand-over-hand mechanism: The ATPase adopts a spiral conformation and makes its way along the substrates’ backbone hand-over-hand by sequential ATP hydrolysis. Thereby, the substrate is in contact with a spiral staircase of conserved aromatic residues that line the central pore. The conformation of these so-called pore loops is functionally coupled to ATP hydrolysis in the same AAA+ domain.

**Figure 2:**
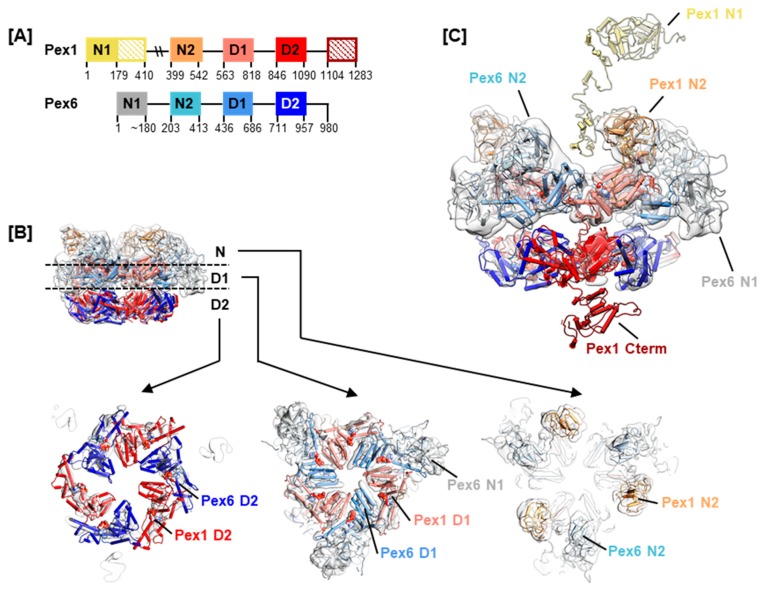
Homology model of the Pex1/Pex6 complex. (**A**) The domain arrangement in Pex1 and Pex6 showing two N-terminal domains termed N1 and N2 as well as the tandem AAA+ ATPase domains termed D1 and D2. Domain boundaries are given as derived from our homology models. (**B**) Fit of the homology model of Pex1/Pex6 into EMDB-6359. The Pex1 N-Terminus (1–410) and C-Terminus (1090–1283) are omitted. The hexameric model is shown as a pipes and planks representation with all domains color coded according to (**A**). A side view of the complex and slabs of the N domains, D1 domains, and D2 domains, as indicated, are shown. (**C**) Model of full length Pex1 protein. Side view of the EM map fitted with the homology model of Pex1/Pex6. One Pex1 protomer is fitted with a Pex1 homology model, lacking only residues 400–409. Pex1 N1 and N2 are connected by an unstructured linker region.

Type II AAA+ ATPases, Pex1 and Pex6 contain two tandem ATPase domains, herein termed D1 and D2 (Figure 2A). In yeast, the hexamer is a trimer of Pex1/Pex6 heterodimers [24,31,32]. EM structures show that the two ATPase domains of the *sc*Pex1/*sc*Pex6 complex form two staggered rings. The N-terminal domains of *sc*Pex6 further pack against the D1 AAA+ ring resulting in an overall triangular shape of the complex [24,31,32] (Figure 2B). Based on interaction studies between the cytoplasmic domain of *sc*Pex15, the yeast homolog of Pex26, and *sc*Pex1/*sc*Pex6, it was proposed that the *sc*Pex1/*sc*Pex6 complex uses a threading mechanism for substrate unfolding [33]. Although Pex5 is considered the cellular substrate of the Pex1/Pex6 complex, the nature of the interaction is unclear. A direct interaction with mono-ubiquitinated Pex5 as well as an adaptor function of Pex15/Pex26 have been suggested [33,34]. The tail-anchored membrane protein Pex26 tethers Pex1/Pex6 to the peroxisomal membrane by interaction between its cytosolic domain and the N-terminal region of Pex6 [33,35]. Aside from Pex26, AWP1 has been described as a Pex1/Pex6 binding adaptor protein in Pex5 recycling [36]. The interaction with cofactors is common for AAA+ ATPases of the NSF and Cdc48-families. The N-terminal domains of NSF, p97, Cdc48, and VAT share a double-Ψ-β-barrel-fold, that has also been reported for *Mm*Pex1 and *sc*Pex1/*sc*Pex6 [31,37]. In p97, cofactors interact with this N-terminal domain to modulate the activity of p97 [38]. In Cdc48, the yeast homolog of p97, a polyubiquitinated substrate is recruited to the central pore by cofactors Npl4 and Udf1 through interaction with the N-terminal domains [39].

Most ZSSD patients carry mutations in the Pex1/Pex6 complex. To date, no structural data on these human AAA+ ATPases are available. However, a wealth of information from low-resolution structures of the *sc*Pex1/*sc*Pex6 complex and homologous AAA+ ATPases exists, which allows to predict the arrangement of the human Pex1/Pex6 complex. In this study, we review the records on Pex1/Pex6 missense mutations found in ZSSD patients. We classify and rationalize them using predictive structural data and sequence analysis methods.

## 2. Results

### 2.1. Selection of Missense Mutations and Generation of Pex1/Pex6 Homolgy Model

Pex1 and Pex6 missense mutations described in PBD patients were collected from publications and databases ClinVar [40], Human Gene Mutation Database (HGMD) [41], and Leiden Open Variation Database (LOVD) [42]. Overall, 133 and 126 mutations were retrieved for Pex1 and Pex6, respectively. The clinical significance of most database records is given as uncertain, and many lack information on the associated clinical condition. Only 37 of these 259 mutations were classified as pathogenic, and for 21 a benign nature was reported. A total of 84 mutations were discussed in publications, of which 17 were biochemically characterized to varying extents. We analyzed mutations that (a) were described in publications, (b) were established as pathogenic or benign, or (c) concerned residues of conserved sequence motifs. This yielded 63 missense mutations for each Pex1 and Pex6 that were mapped onto our homology model of the Pex1/Pex6 complex. In this study, we discussed a total of 69 of these 126 mutations (Table 1). They are distributed throughout the entire complex. Based on the structural motifs, we grouped the mutations into four categories: (I) mutations concerning ATP binding and hydrolysis, (II) mutations concerning substrate interaction, (III) mutations concerning the interaction between Pex1 and Pex6, and (IV) mutations concerning the interaction with cofactors.

Homology models of Pex1 and Pex6 were generated using MODELLER [43], iTASSER [44] and QUARK [45]. A full-length model was obtained for Pex6, while residues 400–409 of Pex1 were missing in the homology model (Figure 2A). Residues 200–409 of Pex1 were consistently predicted to be unstructured [46], and a large portion of this segment is shown as unstructured in our homology model (Figure 2C). Where the well-structured Pex1 N1 domain (residues 1–200) is located in the hexameric complex remains to be determined. Due to the lack of structural data on the human Pex1/Pex6 complex, and given that yeast and human Pex1/Pex6 protein sequences share around 38% identity, we fitted the homology models into the currently best resolved cryo electron microscopy (EM) map of *sc*Pex1/*sc*Pex6, EMDB-6359 (Figure 2B,C) [31]. It is important to bear in mind that type II AAA+ ATPases have been shown to undergo large conformational changes during ATP hydrolysis. The presented hexameric model can only be a snapshot in the reaction cycle of the Pex1/Pex6 complex. Nevertheless, it indicates where conserved motifs and interaction surfaces are positioned in a dynamic assembly.

### 2.2. Mutations Concerning ATP Binding and Hydrolysis

**Figure 3:**
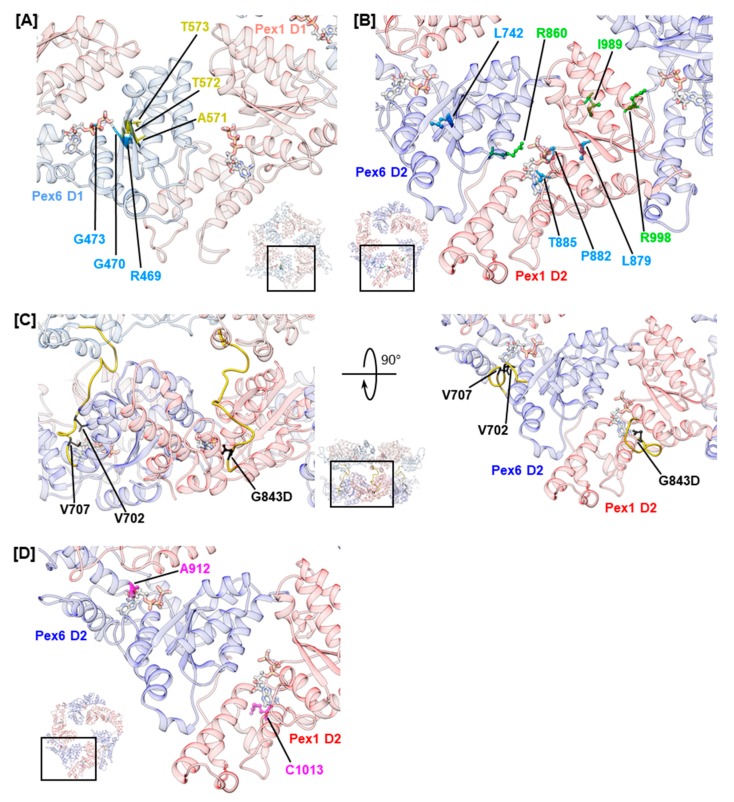
Structural mapping of mutations that concern the ATP binding pocket. Mutations in conserved motifs in D1 (**A**) and D2 (**B**). Amino acids concerned by mutations are shown as sticks and color coded as shown in Figure 2. Insets show the D1 (**A**) and D2 (**B**) ring as a top view with selection of enlarged area. (**C**) Mutations in the D1–D2 linker of Pex1 and Pex6 (right) shown in a front view (left) and top view (right). The linker peptide is colored in yellow and amino acids concerned by mutations are colored in black. The inset shows the front three protomers of the hexamer as a side view with selection of enlarged areas. The mutation G843D is shown as the mutated amino acid. (**D**) Mutations in the α-helical subdomain. Amino acids concerned by mutations are shown in pink. ATP is shown as sticks.

Generally, Pex1/Pex6 D1 domains are less conserved than D2 domains (Figure S1). Most likely, ATP hydrolysis is absent in the D1 ring of Pex1/Pex6 because both peroxins lack arginine residues to stabilize the transition state during ATP hydrolysis (Figure 1D) [71]. The D1 domains of *sc*Pex1 and *sc*Pex6 show similar deviations of conserved motifs and no ATPase activity [24,32]. Yet, ATP binding to the D1 ring is critical for complex formation of *sc*Pex1/*sc*Pex6 [32]. Investigation of Walker A mutations of Pex1 (K605E, K887E) and Pex6 (K476E, K750E) in a mammalian two-hybrid assay and a matrix protein import assay also demonstrated the relevance of ATP binding to D1 and D2 of both peroxins for complex formation [35]. However, we noted that no variations of nucleotide-interacting residues of Pex1 D1 were reported in literature, hinting that ATP binding to this domain might be critical for complex function. This interpretation conforms with findings for the D1 ring of the *sc*Pex1/*sc*Pex6 complex, in which ATP binds only to Pex1 [32]. For the D1 of Pex6, on the other hand, several mutations in the Walker A and sensor 1 motif were reported (Figure 3A). The mutations in the Walker A motif are hPex6.R469W, hPex6.G470A, and hPex6.G473S, which all locate in the p-loop. Residue hPex6.G473 is expected to directly interact with the α- and β-phosphates of the nucleotide as shown for the analogous residue of p97, p97.G521, [72]. Hence, ATP binding to the Pex6 D1 is most likely abolished by substitution of glycine to serine at this position. A similar mode of action is proposed for substitutions hPex6.R469W and hPex6.G470A that precede the nucleotide coordinating residues K476 and T477 of the Walker A motif [35]. The mutations likely alter the geometry of the p-loop and interfere with ATP binding to the Walker A motif. Mutations involving the sensor 1 motif are hPex6.A571T, hPex6.T572I, and hPex6.T573I (Figure 3A). Principally, sensor 1 residues coordinate the attacking water molecule in concert with the Walker B residues. Mutations in the sensor 1 motif consequently diminish ATP hydrolysis [22]. However, the D1 of Pex6 does not harbor a functional Walker B motif. It contains threonine and alanine instead of the conserved aspartate and glutamate residues. Thus, it is unlikely that Pex6 D1 engages in ATP hydrolysis. Subsequently, the pathogenicity of these sensor 1 mutations is not expected to be based on impairment of ATP hydrolysis. In fact, for the pathogenic mutation hPex6.T572I, which precedes the sensor 1 residues hPex6.T573 and hPex6.S574, a temperature-sensitive phenotype was demonstrated in fibroblasts from a homozygous adult patient [57]. This indicates a fold destabilizing effect rather than a catalytically relevant impact, which is further substantiated by the very mild phenotype of the described patient [57].

In contrast to the D1 ring, the highly conserved AAA+ domains in the D2 ring of Pex1/Pex6 harbor all motifs implied in ATP binding and hydrolysis (Figure S2) [24]. Several studies have shown that both D2 domains bind and hydrolyze ATP in *sc*Pex1/*sc*Pex6 [24,32,73]. However, ATP hydrolysis in the *sc*Pex1 D2 domain is not essential for complex function, as cells carrying a Walker B mutation in this domain show wild-type growth on oleate medium [24]. An import assay investigating Walker A and Walker B mutations of human Pex1 and Pex6 demonstrated ATP binding to Pex1 D2 to be critical towards catalase import, while its relevance in ATP hydrolysis was not clear [35]. Both ATP binding and ATP hydrolysis are functionally important for the D2 domain of Pex6 [35]. For Pex6 D2, mutations of the arginine finger residue hPex6.R860, namely hPex6.R860Q and hPex6.R860W, were reported (Figure 3B). Both mutations will most likely impair ATP hydrolysis in the neighboring Pex1 D2 domain. The mutation hPex6.R860Q was described in a patient carrying a R860Q/R601Q Pex6 phenotype who was affected by the mild ZSSD presentation IRD. The mutation hPex6.R860W has been reported for ZSSD patients with an R860W/WT Pex6 genotype [61,62]. Despite the presence of a functional Pex6 allele, these patients present a mild ZSSD phenotype, surviving 8 to 20 years, as they carry an additional mutation in the three prime untranslated region (3′ UTR) of the impaired allele. The 3′UTR mutation deregulates allelic expression, leading to a three-fold to five-fold excess in mRNA levels of the impaired allele, while protein levels of Pex6 in fibroblasts of patients show no anomalies. Yet, ubiquitinated Pex5 clustered at the peroxisomal membrane of patients’ fibroblasts demonstrating an impact on the substrate remodeling capacities of the Pex1/Pex6 complex in these patients [61]. Furthermore, colocalization of both Pex1 and Pex6 at the peroxisomal membrane was noted [61]. These results indicate that mutation hPex6.R860W neither destabilizes the fold of Pex6 nor that of the Pex1/Pex6 complex [35]. The latter contrasts in vitro results obtained for arginine finger mutation *sc*Pex6.R892K that destabilized the assembly of the *sc*Pex1/*sc*Pex6 complex as assessed by size-exclusion chromatography [24]. For the Pex1 D2 domain, a mutation of the second arginine finger residue hPex1.R998Q has been reported (Figure 3B). This residue forms the inter-subunit signaling motif (ISS) together with conserved residue hPex1.D969 and the catalytic glutamate residue of the Walker B motif of the adjacent Pex6 monomer, hPex6.E804 [74]. The mutation has been reported in a patient with an R998Q/I989T Pex1 genotype [53]. The Pex1 protein levels in fibroblasts from this patient were 50%–70% of control fibroblasts, which is considerably higher than the Pex1 protein levels reported for temperature-sensitive mutations hPex1.G843D and hPex1.R798G [14,53]. However, the residual activity of the Pex1/Pex6 complex in all cases is similar. When stained with an anti-SKL antibody, 19% of the fibroblasts carrying the R998Q/I989T Pex1 mutation were peroxisome positive, while 23% and 21% of peroxisome-positive cells were reported for fibroblasts from patients with G843D/G843D and G843D/R798G Pex1 genotypes, respectively [14,53]. This indicates that mutations hPex1.R998Q and hPex1.I989T additionally impair complex function, hinting towards the functional relevance of the second arginine finger residue hPex1.R998 [72]. Furthermore, mutations involving Walker A residues, or their vicinity, have been reported for both Pex1 D2 and Pex6 D2. Mutations (Figure 3B) hPex1.L879S and hPex6.L742P immediately precede the Walker A motif, while mutations hPex1.P882L and hPex1.T885R concern conserved residues within the p-loop. In analogy to Walker A mutations discussed for the D1 domain, these mutations are expected to alter the conformation of the p-loop repositioning the functional lysine and threonine residues. The mutation hPex1.T885R certainly interferes with ATP binding, as the large side chain of arginine most likely occupies the binding site of the adenosine moiety of ATP.

Nucleotide bound to a D2 domain is further in contact with residues of the linker connecting the D2 and D1 domain, which runs across the nucleotide binding pocket (Figure 3C) [72]. The portion of the linker preceding helix α0 of the D2 domain is conserved in p97 from Mammalia, Plantae, and Fungi and in Pex1 and Pex6 (Figure S2) [75]. The D1–D2 linker of Pex1 harbors the most common mutation in ZSSD patients, the mutation hPex1.G843D [13]. It causes reduced Pex1 protein levels of 5–20% in both homozygous and heterozygous patients, which is almost completely rescued at 30 °C [12,14]. It is, thus, commonly referred to as a fold-destabilizing variation, which triggers a temperature-sensitive phenotype. The residue hPex1.G843 is located two amino acids upstream of helix α0 in Pex1 D2 and is predicted to contact the nucleotide. The analogous residue of p97, p97.G480, has been demonstrated to interact with the adenosine moiety of ATP [72]. Thus, impaired ATP binding to Pex1 D2 due to changes in the chemical environment of the ATP-binding pocket might destabilize the fold or vice versa. The analogous Pex6 mutation, hPex6.G708D, has not been described in ZSSD patients to date; nevertheless, it has been characterized. In a complementation assay, Pex6-deficient ZP92 cells expressing hPex6.G708D did not display peroxisomal structures when stained against catalase [69]. In contrast, 50% of Pex1-deficient fibroblasts that expressed hPex1.G843D were peroxisome positive [14]. This difference in impact and clinical relevance may reflect the differing importance of the Pex1 and Pex6 D2 domains in ATP hydrolysis. The mutations of the Pex6 D1–D2 linker described in ZSSD patients are hPex6.V702M and hPex6.V707G (Figure 3D) [40,60]. No phenotype has been reported with an annotation of the mutations. While the latter mutation directly precedes the conserved position hPex6.G708 and is in the vicinity of the nucleotide, the first is removed from the nucleotide binding site. Yet, both mutations concern conserved, hydrophobic positions within the D1–D2 linker [75].

In p97, further contacts between the adenosine moiety of ATP and residues located at the N-terminal end of helices α5 and α7 of the α-helical subdomain have been described [72]. Analogous residues in Pex1 and Pex6 concern reported mutations hPex1.R1013G/H/C (D2, α5) and hPex6.A912V (D2, α7), which might all impact ATP binding. Mutations hPex1.R1013C and hPex6.A912V are classified as pathogenic (Figure 3D).

### 2.3. Mutations Concerning Substrate Interaction

**Figure 4:**
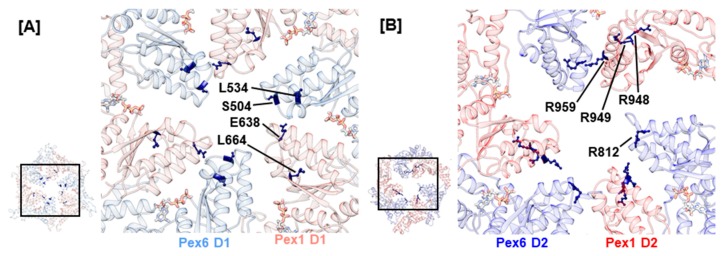
Structural mapping of mutations in pore loops 1 in the D1 ring (**A**) and pore loop 2 in the D2 ring (**B**). Amino acids concerned by mutations are presented as sticks and colored dark blue. Insets show the D1 and D2 ring as a top view with selection of the enlarged area. ATP is visualized as sticks.

The central pore of the AAA+ rings in the Pex1/Pex6 complex is lined by the pore loops 1 and 2, which are embedded into helices α2 and α3, respectively (Figure 1). In analogy to p97, Cdc48, and *sc*Pex1/*sc*Pex6, the D1 ring of the human Pex1/Pex6 complex shows no conserved Ar-Φ-G motif in pore loop 1 of the D1 ring. Noticeably, they contain charged residues: hPex1.K635/R636/E638/N639 and hPex6.E502/S503/S504. Charged residues are also present in the D1 pore loops of ClpB and Hsp104 where they function in substrate engagement and potentially substrate discrimination [76,77]. A recent EM structure of ClpB illustrates that oppositely charged residues of pore loops 1 in the D1 ring of neighboring monomers interact to stabilize the central pore and aid substrate interaction. Furthermore, charge-inverting mutations were shown to substantially lower the affinity of ClpB towards the model substrate casein [29]. Two reported mutations concern the D1 pore loops 1 of the Pex1/Pex6 complex, hPex1.E638A and hPex6.S504N (Figure 4A). They possibly destabilize the central pore as well as interfere with substrate engagement. Another indication that the D1 pore loops are functionally relevant is given by mutation hPex1.L664P, which is located in an α-helical segment preceding pore loop 2 in the Pex1 D1 domain of our model. It causes a temperature-sensitive phenotype and severely impacts the interaction between Pex1 and Pex6, as demonstrated in an immunoprecipitation assay [50]. The introduction of a proline into the α-helix will certainly break this secondary structure element and subsequently alter the conformation of the succeeding pore loop 2. Interestingly, an analogous, pathogenic variation for Pex6 D1 has been described, hPex6.L534P.

The D2 ring of the Pex1/Pex6 complex contains both a conserved Ar-Φ-G motif in pore loop 1 as well as conserved arginine residues flanking pore loop 2, which have been suggested to be critical towards substrate threading [78]. Therefore, we anticipate that these pore loops interact with the substrate. To date, no mutations have been reported for conserved residues hPex1.914Y/915I/916G and hPex6.777Y/778V/779G of the D2 pore loop 1 highlighting their importance in complex function. The conserved arginine residues of D2 pore loop 2, hPex1.R949/R959, and hPex6.R812/R824 are concerned by several mutations (Figure 4B). For both Pex1 and Pex6, arginine substitutions to glutamine or tryptophan have been reported, namely hPex6.R812Q/W, hPex1.R949Q/W, and hPex1.R959Q/W. Furthermore, the arginine residue hPex1.R948, which directly precedes hPex1.R949, can be substituted to glutamine or proline. The pathogenic nature of mutation hPex1.R949Q was shown in fibroblasts of a ZS patient with a G843D/R949Q Pex1 genotype [12]. Although Pex1 protein levels were determined at 50%, when stained against catalase, no punctuate signals were evident in fibroblasts from the patient. This phenotype was, furthermore, not rescued at 30 °C. Consequently, the patient died at 3 months of age. Peroxisomal structures were also absent in peroxisome-deficient CHO cells, which were complemented with hPex6.R812Q and hPex6.R812W [66]. An associated patient harboring the hPex6.R812W mutation alongside a splice variant also displayed the severe ZS phenotype. This indicates that mutations of the conserved arginine residues of the D2 pore loop 2 impact Pex1/Pex6 function rather than destabilize fold or complex formation.

### 2.4. Mutations Concerning the Interaction between Pex1 and Pex6

**Figure 5:**
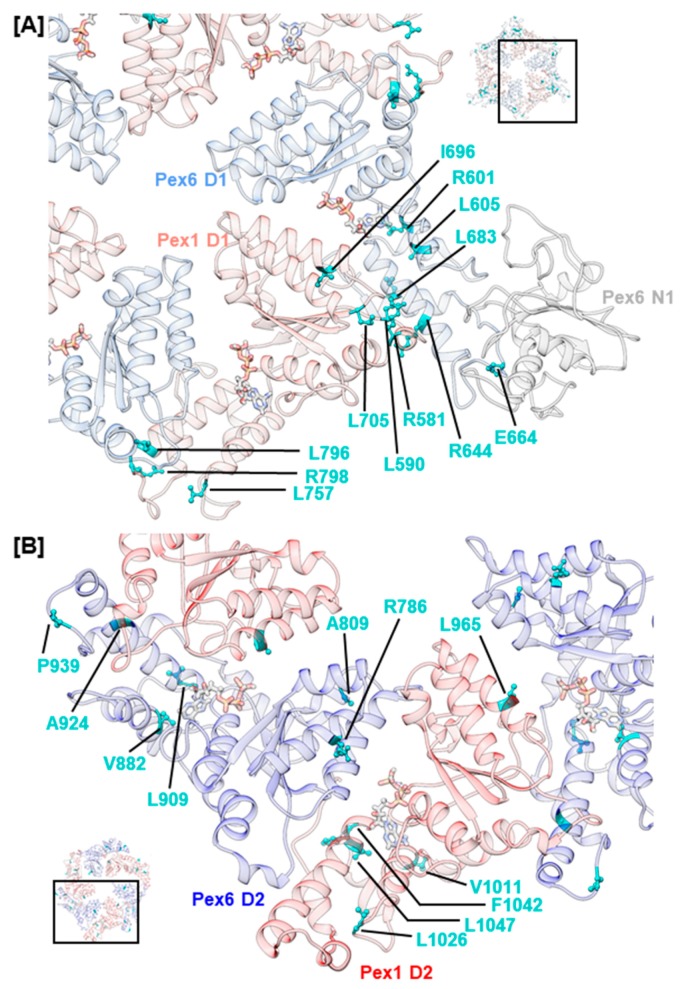
Structural mapping of mutations that concern the interaction between Pex1 and Pex6 in the D1 ring (**A**) and the D2 ring (**B**). Concerned amino acids and ATP are shown as sticks. Insets show the D1 ring and D2 ring as top views with selection of the enlarged area.

The interaction between Pex1 and Pex6 is predominantly mediated by helices α2–α4 in the α/β-Rossmann fold of one monomer and helices α5–α8 of the α-helical subdomain of the counterclockwise-positioned monomer. Noticeably, few mutations concerning side chains that project towards the Pex1/Pex6 interface were recorded in helices α2–α4 (Figure 5). Most likely, mutations of this type directly impede interaction with the adjacent monomer by altering charge, hydrophobicity, or stability of secondary structure elements. Examples are mutations hPex1.L965P (D2, α3) and hPex6.R786W (D2, α3). The first mutation is classified as pathogenic and likely induces a break in helix α3 of Pex1 in our model, while the latter introduces a hydrophobic, sterically demanding residue into a charged environment. The remaining mutations in helices α2–α4 and nearby loop regions concern hydrophobic residues, whose sidechains are buried in our model. Most of them are classified as benign or retain chemical properties. An example is hPex1.L705W (D1, α3–β4), whose clinical significance is uncertain. In 58% of Pex1-deficient fibroblasts that expressed hPex1.L705W alongside pEGFP-SKL, punctuate signals were observed that represented peroxisomes [3]. In two patients 29 and 31 years old with an L705W/I700YfsX42 Pex1 genotype, the mild HS phenotype was diagnosed. Examples of benign mutations located in helices α2–α4 are the variations hPex1.I696M (D1, α3) and hPex6.A809V (D2, α3).

For the α-helical subdomain of the D1 domain of both Pex1 and Pex6, mutations are described that involve surface-exposed residues located at the C-terminal end of α7, which packs against α0 of the adjacent monomer. The mutations, namely hPex1.L796P (D1, α7), hPex1.R798G (D1, α7–α8), hPex6.R644W (D1, α7), and hPex6.E664D (D1, α7–α8), alter the charge at the interaction surface in our model and possibly hamper the interaction between the peroxins (Figure 5A). Mutations hPex1.R798G and hPex6.E664D are both located at the loop region succeeding α7 and might have a fold-destabilizing effect as the conformation of loops can be stabilized by electrostatic interactions. Our model suggests respective interactions between hPex1.R798 and hPex1.D813 (D1, α8) as well as hPex6.E664 and hPex6.K651 (D1, α7–α8) or hPex6.N652 (D1, α7–α8). The expression of hPex1.R798G in Pex1-deficient fibroblasts led to approximately 50% peroxisome-positive cells [14]. A temperature-dependent phenotype was also demonstrated on fibroblasts from a heterozygous patient displaying a G843D/ R798G Pex1 genotype. After staining with anti-catalase or anti-SKL antibodies, punctuate signals were evident in 83% and 91% of fibroblasts after cultivation at 30 °C, respectively. Thus, mutation hPex1.R798G was suggested to impact protein folding. It should be noted that this phenotype may also relate to mutation hPex1.G843D [14]. Furthermore, mutations that concern helix α0 of Pex1 D1, such as hPex1.R581P (D1, α0) and hPex1.L590R (D1, α0), have been reported. The pathogenic nature of mutation hPex1.R581P, which is expected to destabilize α0, has been demonstrated in a complementation assay. Only 23% of Pex1-deficient fibroblasts that expressed hPex1.R581P alongside pEGFP-SKL displayed punctuate signals that represented peroxisomes [3].

Interestingly, few mutations were uncovered, which concern the C-terminal part of α7 and α0 in the D2 ring of the Pex1/Pex6 complex (Figure 5B). One such mutation, hPex6.A924S (D2, α7), is classified as benign (ClinVar), though it concerns a surface-exposed residue in our model and alters hydrophobicity. Moreover, several benign variations were identified for the α-helical subdomain. These are hPex1.L757F (D1, α5–α6), hPex1.V1011M (D2, α5), hPex6.V882I (D2, α5), hPex6.A924S (D2, α7), and hPex6.P939Q (D2, α8). The remainder of mutations reported for the α-helical subdomain in Table 1 are expected to have fold-destabilizing properties based on their distinct localization and nature of the substitution. The mutations hPex1.L1026P (D2, α5–α6), hPex1.F1042V (D2, α6–α7), hPex1.L1047P (D2, α7), hPex6.L605R (D1, α5), hPex6.L683P (D1, α8), and hPex6.L909Q (D2, α6–α7), for example, concern hydrophobic residues whose sidechains are buried within the hydrophobic core of the α-helical bundle in our model. Besides the destabilizing effect introduced by the removal of the hydrophobic residue, the introduction of proline residues in helical segments generally disturbs the secondary structure and destabilizes them. The common founder mutation hPex6.R601Q (D1, α5) that is located within the second turn of α5 could also destabilize the α-helical subdomain, as buried polar residues provide fold-stabilizing hydrogen bond interactions with backbone atoms [79]. Its clinical significance is currently unclear, with two depositors attributing a benign nature while five other depositors considered this mutation to be pathogenic.

Yeast two-hybrid assays further indicated a relevance of the Pex1 C-terminus in Pex1/Pex6 oligomerization and complex stability, as the C-terminal truncation Pex1(1-1216) displayed a diminished interaction with Pex6 [35]. Several predictors of protein disorder identify the last approximately 200 residues of Pex1 to be disordered [46]. However, protein structure prediction using the iTASSER server produced a structured model of the Pex1 C-terminus (Figure 2C). It is impossible to predict where exactly the Pex1 C-terminus is located in the hexameric assembly. The relevance of the Pex1 C-terminus is, however, illustrated by deletions hPex1.Q1231HfsX3 and hPex1.W1250X, both described in homozygous patients. The patient affected by the first mutation presented a severe ZS phenotype and died at two months of age [11]. The second variation has been described in two individuals of 12 and 16 years at assessment, who presented a mild HS phenotype [3]. This suggests a functional relevance of C-terminal residues succeeding position 1231, although the final 30 residues of Pex1 seem less important for complex function or integrity. Whether the C-terminus of Pex1 is exclusively relevant for complex stability is unknown. In p97, the C-terminus interacts with the PUB (peptide N-glycanase/ UBA or UBX-containing protein) domain present in several cofactors and further modulates ATPase activity of the D2 ring [72].

Generally, we noted that 13 of the 21 variations classified as benign are located in parts of the Pex1/Pex6 model, which are relevant for Pex1/Pex6 interaction. Six benign variations are located at the α-helical subdomain, five concern the interaction surface formed by helices α2–α4, and three concern the C-termini of Pex1 or Pex6. The remaining eight benign variations are located in the N-termini of Pex1 and Pex6 including the N2-D1 linker. Five of these eight benign variations are in the N-terminus of Pex1.

### 2.5. Mutations Concerning Interactions with Cofactors

**Figure 6:**
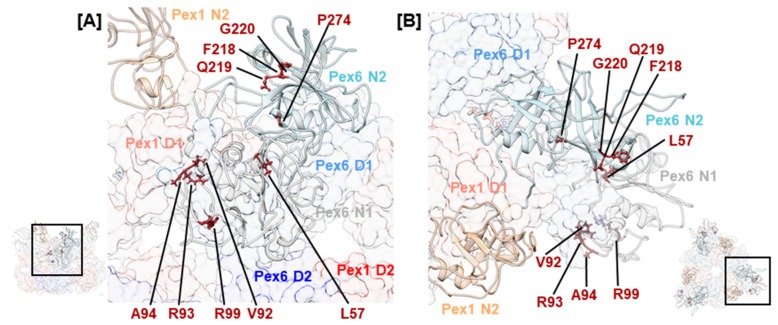
Structural mapping of mutations that concern the N-terminal domains N1 and N2 of Pex6. Concerned amino acids and ATP are shown as sticks. Insets show a side view of the hexamer with Pex6 in the front position (**A**) and a top view of the Pex1/Pex6 complex (**B**). Enlarged areas are indicated.

Pex26 has been established as a cofactor that interacts with the N-terminal domain of Pex6 [33,50]. Three consecutive mutations, hPex6.F218L, hPex6.Q219P, and hPex6.G220V, in the Pex6 N2 domain were noted (Figure 6). The first mutation is classified as pathogenic, while the latter two are of uncertain significance. They concern residues whose sidechains are oriented towards the groove between the N-terminal and C-terminal subdomain of Pex6 N2 in our model. In the N-terminal domain of p97, this groove is an interaction site for many cofactors [38]. Superimposition of Pex6 N2 with the N-terminal domain of p97 demonstrates a homology between the above-mentioned residues and p97.F52, p97.R53, and p97.G54 (Figure S3). The arginine residue p97.R53 directly interacts with the p97/valosin containing protein-interacting motif, an α-helical structure that is present in several cofactors of p97 [80]. Structural predictions also identify an α-helical structure in the supposedly folded core of Pex26 [33]. Thus, the clinical relevance of mutations hPex6.F218L, hPex6.Q219P, and hPex6.G220V indicate a potential interaction site between Pex6 and Pex26 at the groove of the Pex6 N2 domain. In yeast on the other hand, the binding site of Pex15 on Pex6 was located between the N1 and N2 domain of Pex6 [33]. Since the cytosolic domain of Pex26 cannot complement the cytosolic domain of Pex15 in a yeast-based complementation assay [81], differences in the binding mode of both peroxins are expected.

A cluster of mutations is observed in the Pex6 N1 domain. Mutations hPex6.V92G, hPex6.R93G, hPex6.A94E/K/L, and hPex6.R99L/W locate to an arginine-rich segment of the Pex6 N1 domain (Figure 6). According to our homology model, these residues are positioned in a surface-exposed loop, indicating another potential interaction site. Many of the remaining mutations in Table 1 locate to the N-termini of the Pex1/Pex6 complex and indicate a fold-destabilizing effect based on the position and the nature of the substitution. For instance, a temperature-sensitive phenotype has been demonstrated for hPex6.L57P: After staining with an anti-catalase antibody of Pex6-deficient CHO cells that expressed hPex6.L57P, 10% of cells displayed punctuate signals when cultivated at 37 °C, while 80% did so when cultivated at 30 °C. [69]. Fold destabilization has also been demonstrated for the frequent mutation hPex6.P274L that concerns a residue within the Nc subdomain of the N2 domain. Cultivation of fibroblasts of an individual with a P274L/E439fsX3 Pex6 genotype in the presence of 20 mM arginine increased the number of cells that displayed punctuate signals after staining against catalase by approximately 20% [15]. Interestingly, this variant of Pex6 does not complement in Pex6-deficient fibroblasts [3], which indicates a profound effect of the mutation on the fold of the Pex6 N2 domain. A similar example is mutation hPex1.V92L located in the N1 domain of Pex1. Although the substitution of valine with leucin is conservative, a homozygous ZSSD patient with a postnatal survival of 1 year and 11 months was reported [11].

## 3. Discussion

Clinically relevant missense mutations for the peroxisomal AAA+ ATPases Pex1 and Pex6 do not cluster to specific regions of the peroxins but are distributed throughout the complex. At this stage, due to the lack of structural data of the Pex1/Pex6 complex from *Homo sapiens*, the location of individual mutations can only be predicted. The herein presented structural mappings of a subset of mutations on homology models in combination with general knowledge on catalytic and mechanistic features of AAA+ ATPases can facilitate characterization of each mutation. In this review, we do not only predict the location of individual mutations in the Pex1/Pex6 hexamer, but we relate this structural mapping to known sequence motifs of AAA+ proteins, which have been shown to confer specific functions [22] and are present in Pex1/Pex6. Thus, Pex1/Pex6 mutations can be roughly grouped according to their structural and functional significance.

For many mutations—among them the most frequently encountered in patient cohorts—a temperature-sensitive phenotype has either been demonstrated or is expected. Fold destabilization is a common pathogenic factor of missense mutations throughout hereditary diseases since many natively folded proteins occupy a narrow niche of the energy landscape [82]. An in silico investigation into missense mutations of multidomain proteins associated with inherited eye disease estimated that 80% of clinically relevant missense mutations exerted a fold destabilizing effect [83]. For the fold destabilizing mutation hPex1.G843D, an increase in peroxisomes was demonstrated in fibroblasts of affected patients when cultured in the presence of arginine, betaine, and flavonoids [15,16,17]. These substances, thus, stabilize the Pex1/Pex6 assembly. Without detailed knowledge on where the molecules bind on Pex1/Pex6, it is impossible to predict whether their effect would also apply to other fold-destabilizing mutations. However, it is plausible that ZSSD patients carrying temperature-sensitive, fold-destabilizing mutations in either Pex1 or Pex6 are amenable to chaperone treatment with small compounds and will benefit from further exploration of this approach.

At the same time, several clinically relevant mutations involve highly conserved residues in functional motifs of Pex1 or Pex6. Biochemical data on some of these mutations verified the expected profound impact on Pex1/Pex6 function beyond mere fold destabilization. The presence of mutations involving functionally relevant residues was initially surprising. For the ubiquitous AAA+ ATPase p97, which is also implied in hereditary diseases, no variations of conserved structural motifs have been reported [84]. On the other hand, deletion of p97 appears to be lethal in the embryonic stage [85]. In contrast, homozygous patients carrying the mutation hPex1.I700YfsX42, which results in nondetectable Pex1 protein levels, were reported with postnatal survival of 2 to 12 months [12]. This suggests a lower system relevance of the Pex1/Pex6 complex compared to p97.

It is questionable whether patients affected by missense mutations of functionally relevant residues benefit from fold-stabilizing agents as therapeutics. They may, however, benefit from the development of drugs that target pexophagy [86]. This process has recently emerged as a putative therapeutic target due to a reinterpretation of the Pex1/Pex6 complex from a transporter-recycling gear to a pexophagy-limiting gear [87,88]. Autophagy inhibitors were demonstrated to partially rescue the phenotype of Pex1-devoid fibroblasts [87]. The development of such drugs could not only be beneficial to ZSSD patients affected by missense mutations of varying severity but also to patients with class I mutations.

## 4. Materials and Methods

### 4.1. Homology Modeling

Homology models of Pex1 (UniProt: O43933) and Pex6 (UniProt: Q13608) were constructed with MODELLER within HHPred [43,89], the iTASSER server [44], and the QUARK server [45]. Queries were submitted to the iTASSER and QUARK server using default settings without further restraints (Table 2). Separate models belonging to the same protein were connected by superimposition of corresponding secondary structure elements in Chimera [90] using the MatchMaker tool. Where necessary, one model was translated using a custom R script to achieve matching coordinates of either a backbone C or Cα atom in both models. This R script was further utilized to correct residue and atom IDs as well as chain information.

The assembled models of Pex1 and Pex6, comprising residues 1–399/410–1283 and 1–980, respectively, were fitted into the cryo electron microscopy density of EMDB-6359 (*sc*Pex1/*sc*Pex6, ATPγS, 7.2 Å, [31]) corresponding to *sc*Pex1 or *sc*Pex6, respectively. Fitting was done with Flex-EM [91] in MD mode for 10 iterations with a resolution of 7.2 and an atom displacement cutoff of 0.2. The individual domains N1, N2, D1, and D2 were manually defined as rigid bodies. In a second round of Flex-EM fitting, the α/β Rossmann fold and the α-helical subdomain of each ATPase domain were defined as individual rigid bodies to allow for rotation between the subdomains. Cross-correlation between the assembled Pex1/Pex6 model and EMDB-6359 was determined in Chimera using the fitmap command with option res 7.2.

### 4.2. Sequence Alignments and Conservation Score

Pex1 (UniProt: O43933) and Pex6 (UniProt: Q13608) were each submitted to pBLAST against the nr-database and the RefSeq database using default parameters with a maximum output of 20,000 sequences each. Using a custom R script, the raw sequence data were curated:

- sequences whose name indicated a Pex1 or Pex6 sequence were retrieved;
- sequences whose name indicated a putative annotation or a low quality were excluded;
- doubled or contained sequences were excluded; and
- one sequence per species was kept, generally, the sequence referring to isoform 1 or a RefSeq sequence.

This procedure yielded 301 and 454 sequences for Pex1 and Pex6, respectively. Multiple sequence alignments were produced with Clustal Omega [92] using default parameters and visualized in JalView [93]. Where necessary, ill-fitting sequences were manually removed from the stack. The conservation score was directly adopted as presented by JalView.

### 4.3. Analysis of Mutations Described in Peroxisome Biogenesis Disorders (PBDs) Patients

Variations described in PBD patients for Pex1 and Pex6 were collected from publications and databases ClinVar [40], HGMD [41], and LOVD [42]. Information on the clinical significance and clinical condition of each record were adopted from the ClinVar database. For records without or incomplete ClinVar entries, the information was deduced from respective publications or databases. Variations that were reported from either a homozygous patient or were provided with appropriate biochemical data were interpreted as likely pathogenic. Only variations that were either (1) described in publications, (2) were established as pathogenic or benign, or (3) concerned residues of conserved sequence motifs were further analyzed. Visualization, structural mapping, and analyses were performed in Chimera.

## Figures and Tables

**Table 1:** Mutations of the Pex1/Pex6 complex reported from Zellweger syndrome spectrum disorder (ZSSD) patients that are discussed in this study. Mutations were collected from databases ClinVar, Human Genome Mutation Database (HGMD), and Leiden Open Variation Database (LOVD) and publications. The mutations are grouped into four categories: (**I**) mutations concerning ATP binding and hydrolysis, (**II**) mutations concerning substrate interaction, (**III**) mutations concerning the interaction between Pex1 and Pex6, and (**IV**) mutations concerning the interaction with cofactors.

**I—Mutations Concerning ATP Binding and Hydrolysis**										
**Domain**	**Putative SSE ^a^**	**Concerned Motif ^b^**	**Exon**	**cDNA Level**	**Protein Level**	**Clinical Significance ^c^**	**Condition**	**Patient ^d^**	**Database**	**Publication**
Pex1 D1–D2			15	c.2528G>A	p.G843D	p [40]		p.G843D/p.G843D, 2 years 9 months (d)/9 years (d), n/a [12]; p.G843D/p.G843D, 8–45 years (a), n/a [12]	[40,41,42]	[10,12,14,18,47,48,49,50,51,52]
Pex1 D2	β1	(Walker A)	16	c.2636T>C	p.L879S	u	ZSSD [13]		[41]	[13,18]
	β1–α1	Walker A	16	c.2645C>T	p.P882L	u	n/a		[40]	
	α1	Walker A	16	c.2654C>G	p.T885R	u	ZSSD [13]		[41]	[13]
	α4	**ISS**	19	c.2993G>A	p.R998Q	p(−) [53]	ZSSD [53]	p.I989T/p.R998Q, 127 months (d), ZS [53]	[41,42]	[53]
	α5		20	c.3038G>A	p.R1013H	u	ZSSD	p.R1013H/p.S1096X, 4 months (d), NALD [11]	[40,41]	[11]
	α5		20	c.3037C>G	p.R1013G	u	ZSSD [54]	p.R1013G/p.I700Yfs42X, <10 months (d), n/a [54]	[41]	[54]
	α5		20	c.3037C>T	p.R1013C	p(−) [55]	ZSSD [55]	p.R1013C/p.R1013C, 1 month (d)/20–36 months (a), n/a (5 patients) [55]	[41]	[55]
Pex6 D1	β1	(Walker A)	6	c.1405C>T	p.R469W	u	n/a		[40]	
	β1–α1	Walker A	6	c.1409G>C	p.G470A	u	ZSSD		[40]	
	β1–α1	Walker A	6	c.1417G>A	p.G473S	u	n/a		[40]	
	β4	(sensor 1)	8	c.1711G>A	p.A571T	u	ZSSD [56]		[41,42]	[56]
	β4–α4	(sensor 1)	8	c.1715C>T	p.T572I	p [40]	ZSSD [57], HS [58]	p.T572I/p.T572I, adult (a), IRD [57]; p.T572I/splice variant, 17 months (d), ZS–NALD [57]; p.T572I/p.T572I, 22 years (a), HS [58]; p.T572I/p.T572I, 35 years (a), HS [58]	[40,41,42]	[57,58]
	β4–α4	sensor 1	8	c.1718C>T	p.T573I	u	ZSSD		[40,41]	[59]
Pex6 D1–D2			11	c.2104G>A	p.V702M	u	n/a		[40]	
			11	c.2120T>G	p.V707G	u	ZSSD [60]		[41,42]	[60]
Pex6 D2	β1	(Walker A)	11	c.2225T>C	p.L742P	u	ZSSD		[40,41,42]	[56]
	α4	**arginine finger**	14	c.2578C>T	p.R860W	p [61]	ZSSD	p.R860W/p.P285A, n/a, IRD [42]; p.R860W/WT, n/a, n/a [62]; p.R860W/WT, 8–20 years (d), ZSSD [61]	[41,42]	[61,62]
	α4	**arginine finger**	14	c.2579G>A	p.R860Q	u	ZSSD [62]	p.R860Q/p.R601Q, n/a, IRD [42,62]	[41,42]	[62]
	α7		16	c.2735C>T	p.A912V	p(−) [42]	ZSSD [42]	p.A912V/p.A912V, n/a, NALD [42]	[42]	[18]
**II—Mutations Concerning Substrate Processing**										
**Domain**	**Putative SSE ^a^**	**Concerned Motif ^b^**	**Exon**	**cDNA Level**	**Protein Level**	**Clinical Significance ^c^**	**Condition**	**Patient ^d^**	**Data Base**	**Publication**
Pex1 D1	α2	pore loop 1	12	c.1913A>C	p.E638A	u	n/a		[40]	
	α3	(pore loop 2)	12	c.1991T>C	p.L664P	p(−) [63]	ZSSD	p.L664P/p.634del690, 2 months (d), ZS [63]	[40,42]	[11,63]
Pex1 D2	α3	(pore loop 2)	18	c.2843G>A	p.R948Q	u	ZSSD [62]	Pex1:p.R948Q/WT, Pex26:p.R98W/p.R98W, n/a, n/a [62]	[40,41]	[62]
	α3	(pore loop 2)	18	c.2842C>T	p.R948W	u	ZSSD		[40]	
	α3	**pore loop 2**	18	c.2846G>A	p.R949Q	p(−) [12]	ZSSD	p.G843D/p. R949Q, 3 months (d), ZS [12]; p.R949Q/?, n/a, ZS [11]	[40,41,42]	[11,12]
	α3	**pore loop 2**	18	c.2845C>T	p.R949W	u	ZSSD	p.R949W/p.V336A/p.S555P, n/a, n/a [13,18]; p.R494W/p.H678fsX3, 2 months (a), ZS [64]	[40,41]	[13,18,64]
	α3	**pore loop 2**	18	c.2876G>C	p.R959P	u	ZSSD		[40]	
	α3	**pore loop 2**	18	c.2876G>A	p.R959Q	u	n/a		[40]	
Pex6 D1	α2	pore loop 1	7	c.1511G>A	p.S504N	u	n/a		[40]	
	α3	(pore loop 2)	7	c.1601T>C	p.L534P	p [40]	ZSSD		[40]	[65]
Pex6 D2	β3	**pore loop 2**	13	c.2434C>T	p.R812W	p [66]	ZSSD [66]	p.R812W/p.R601Q, 3 years 6 months (a), nonclassical ZS [42,66]	[41,42]	[66]
	β3	**pore loop 2**	13	c.2345G>A	p.R812Q	p(−) [40]	ZSSD [66]	p.R812Q/splice variant, n/a, ZS [66]	[40,41,42]	[66]
**III—Mutations Concerning the Interaction Between Pex1 and Pex6**										
**Domain**	**Putative SSE ^a^**		**Exon**	**cDNA Level**	**Protein Level**	**Clinical Significance ^c^**	**Condition**	**Patient ^d^**	**Data Base**	**Publication**
Pex1 D1	α0		10	c.1742G>C	p.R581P	p [40]	HS [3]	p.R581P/splice variant, 19 years (a), HS [3]; p.R581P/p.I700YfsX42, 24 years (a), HS [3]	[40]	[3]
	α0		10	c.1769T>G	p.L590R	p(−) [62]	ZSSD [62]	p.L590R/p.L590R, n/a, n/a [62]	[41]	[62]
	α3		13	c.2088A>G	p.I696M	b [40]	n/a		[40,42]	[51]
	α3–β4		13	c.2114T>G	p.L705W	u	HS [3]	p.L705W/p.I700YfsX42, 29 and 31 years (a), HS [3]	[40]	[3]
	α5–α6		14	c.2271G>C	p.L757F	b [40]	n/a		[40]	
	α7		14	c.2387T>C	p.L796P	u		p.L796P/p.S304CfsX4, 5 months (a), ZS [11]	[41]	[11]
	α7		14	c.2392C>G	p.R798G	u		p.R798G/p.G843D, 15 months (d), ZS [14]	[41,42]	[14,53]
Pex1 D2	α3		18	c.2894T>C	p.L965P	p(−) [40]	n/a		[40]	
	β4–α4		19	c.2966T>C	p.I989T	c [40]	ZSSD	p.I989T/p.R998Q, 127 months (d), ZS [53]; p.I989T/p.p.Q598TfsX11, 45 years (a), HS [58]	[40,41,42]	[53,58]
	α5		20	c.3031G>A	p.V1011M	c [40]	ZSSD		[40]	
	α5–α6		20	c.3077T>C	p.L1026P	p(−) [67]	ZSSD	p.L1026P/p.L1026P, 6 years (a)/12.5 years (d), NALD [67]	[40]	[67]
Pex1 C-term			23	c.3691_3694delCAGT	p.Q1231HfsX3	p(−) [11]	ZSSD [11]	p.Q1231HfsX3/p.Q1231HfsX3, 2 months (d), ZS [11]		[11]
			23	c.3750G>A	p.W1250X	p(−) [68]	HS	p.W1250X/p.W1250X, 12 and 16 years (a), HS [68]		[68]
Pex6 D1	α5		8	c.1802G>A	p.R601Q	c [40]	ZSSD, HS	p.R601Q/p.L614RfsX5, 21 years (a), HS [3]; p.R601Q/p.R860Q, n/a, IRD [62]; p.R601Q/p.R812W, 3 years 6 months (a), nonclassical ZS [42,66]	[40,41,42]	[3,56,58,62,66]
	α5		8	c.1814T>G	p.L605R	u	ZSSD [56]		[41,42]	[56]
	α7		9	c.1930C>T	p.R644W	p [40]	HS	p.P274L/p.R644W, 21 years (a), HS [3]; |p.P274L/p.R644W, 16 years (a), HS [3]	[40]	[3]
	α7–α8		10	c.1992G>C	p.E664D	u	ZSSD [56]		[41,42]	[13,18]
	α8		10	c.2048T>C	p.L683P	u	ZSSD [60]		[41,42]	[60]
Pex6 D2	α2		12	c.2356C>T	p.R786W	u	ZSSD [56]		[40,41,42]	[56]
	α3		13	c.2426C>T	p.A809V	b [40,56]	ZSSD [56]	splice variant/p.A809V/p.I845T, adult (a), n/a [57]	[40,41,42]	[56,57,60]
	α5		15	c.2644G>A	p.V882I	b [40]	n/a		[40,42]	
	α6–α7		16	c.2726T>A	p.L909Q	u	ZSSD [56]		[41,42]	[56]
	α7		16	c.2770G>T	p.A924S	b(−) [40]	n/a		[40,42]	
	α8		17	c.2816C>A	p.P939Q	b [56,62]	n/a		[40,42]	[56,62]
**IV—Mutations Concerning the Interaction with Cofactors**										
**Domain**	**Exon**			**cDNA Level**	**Protein Level**	**Clinical Significance ^c^**	**Condition**	**Patient ^d^**	**Data Base**	**Publication**
Pex1 N-term	3			c.274G>C	p.V92L	p(−) [11]	ZSSD [11]	p.V92L/p.V92L, 1 year 11 months (d), nonclassical ZS [11]	[41]	[11]
Pex6 N1	1			c.170T>C	p.L57P	p(−) [69]	ZSSD [69]	p.L57P/p.L57P, n/a, NALD [69]	[41,42]	[69]
	1			c.275T>G	p.V92G	p [40]	ZSSD	p.R92L/p.R601Q, 12 years (a), HS [58]	[40]	[58]
	1			c.277C>G	p.R93G	u	ZSSD [56]		[41,42]	[56]
	1			c280G>C	p.A94P	p(−) [70]	ZSSD [70]	p.A94P/p.A94P, 6 years (d), mild ZS [70]		[70]
	1			c.281C>T	p.A94E	u	ZSSD [51]		[41]	[51]
	1			c.281C>A	p.A94K	u	ZSSD [51]		[42]	[51]
	1			c.296G>T	p.R99L	p [40]	ZSSD	p.R99L/R601Q, 7 years (a), HS [58]	[40]	[58]
Pex6 N2	1			c.654C>G	p.F218L	p(−) [58]	ZSSD	p.F218L/p.R601Q, n/a, HS [58]	[40]	[58]
	1			c.656A>C	p.Q219P	u	ZSSD		[41,42]	[56]
	1			c.659G>T	p.G220V	u	ZSSD		[40,41,42]	[56]
	1			c.821C>T	p.P274L	p [40]	ZSSD [62], HS [3]	P274L/R644W, 21 years (a), HS [3]; P274L/R644W, 16 years (a), HS [3]; P274L/E439fsX3, n/a, n/a [15]; P274L/splice variant, n/a, n/a [62]	[40,41,42]	[3,15,51,62]

^a^ Secondary structure element (SSE) of the AAA+ ATPase domain, in which the mutation is located according to our homology model. ^b^ For mutations concerning residues in conserved structural motifs or their vicinity, the motif is given as follows: (motif), the concerned residue is positioned near the motif; motif, the concerned residue is part of the conserved motif; **motif**, the concerned residue is part of the conserved motif and interacts with the nucleotide. ^c^ Clinical significance of a mutation is given as follows: u, uncertain significance; b, benign; b(−), likely benign; p, pathogenic; p(−), likely pathogenic; and c, conflicting interpretations. Information on clinical. Significance was adopted from the ClinVar database. For records without or incomplete ClinVar entries, the information was deduced from respective publications or other databases. Variations that were reported from either a homozygous patient or were provided with appropriate biochemical data were interpreted as likely pathogenic. For mutations whose clinical significance is established, the respective reference is given. ^d^ Patients that were described in combination with particular mutations are given with their genotype, their age of death (d) or age at last assessment (a), and the clinical phenotype. Information not available is marked as n/a. ISS, inter-subunit signaling motif; ZS, Zellweger syndrome; IRD, infantile Refsum disease; HS, Heimler syndrome; NALD, neonatal adrenoleukodystrophy.

**Table 2:** Homology models of Pex1 and Pex6 generated and used in this study in conjunction with the individual domains of Pex1 and Pex6 they encompass.

**Domain**	**Residues ^b^**	**Model ^a^**	**Resource ^a^**	**Range ^a^**	**Templates ^a^**
Pex1 N1	1–179	1	iTASSER	1–399	1WLF (88%), 3HU3 (13%), 3JC8 (9%), 4KO8 (12%), 1TZL (8%)
	178–399				
Pex1 N2	410–542	2	MODELLER	1–1238	1WLF (89%), 5E7P (32.9%), 5VC7 (38.5%), 5G4F (32.9%), 5KWA (36.3%), 6MAT (30.6%), 6MCK (36.1%)
Pex1 D1	563–818				
Pex1 D2	846–1090	3	iTASSER	838–1238	5G4F (49%), 6MAT (23%), 6AZ0 (23%), 5VC7 (48%)
Pex1 C-terminus	1104–1283				
Pex6 N1	1 to about 180	4	QUARK	1–200	
Pex6 N2	191–413	5	MODELLER	200–400	4RV0 (18.9%), 5B6C (15.8%), 1CZ4 (15.5%), 5G4F (13.6%), 1WLF (17.2%), 1ZC1 (13.8%), 2YUJ (12.3%), 5E7P (16.5%), 1QCS (10.1%), 5FTJ (15.1%)
Pex6 D1	440–687	6	MODELLER	1–980	5FTJ (27.6%), 5G4F (28.2%), 5E7P (25.3%), 6MCK (33.1%), 6MAT (30.8%)
Pex6 D2	709–958				
Pex6 C-terminus	963–980				

^a^ Individual homology models are identified by their number, the resource that was used to construct them, and the sequence range of the query protein that was submitted. The structures employed by MODELLER or iTASSER are identified via their PDB code, the sequence identity between the template and the query in the aligned region is given in brackets. ^b^ The residues encompassed by each domain.

## Supplementary Materials

Supplementary materials can be found at https://www.mdpi.com/1422-0067/20/15/3756/s1.

## Abbreviations

NTD	N-terminal domain
ZSSD	Zellweger syndrome spectrum disease
ZS	Zellweger syndrome
HS	Heimler syndrome
EM	Electron microscopy
PBD	Peroxisome biogenesis disorder
NSF	*N*-ethylmaleimide sensitive factor
