# Supplementary material for: Structural Mapping of Missense Mutations in the Pex1/Pex6 Complex
Source: Int J Mol Sci. 2019 Aug 1;20(15):3756. doi: 10.3390/ijms20153756 (PMC6696164; doi:10.3390/ijms20153756)
Supplement: Supplementary file 1 [file ijms-20-03756-s001.zip › ijms-565052-SI.docx]

**Figure S1.** Comparison of sequences of human and yeast (*Saccharomyces cerevisiae*) Pex1 and Pex6 and with homologous proteins p97 (*Homo sapiens*), VAT (*Thermoplasma acidophilum*) and NSF (*Homo sapiens*). The position of conserved sequence motifs of the AAA+ ATPase domains D1 and D2 are highlighted by boxes and annotated above the alignment. The boundaries of domains N1, N2, D1 and D2 are indicated by flanking arrows. The boundaries of the N1 and N2 domains are estimated based on the homology models of Pex1 and Pex6. The alignment was prepared with Clustal Omega using default parameters and visualized in JalView.

**Figure S2.** Conservation of the D1–D2 linker of Pex1 (left) and Pex6 (right). Multiple sequence alignments of homologs from species *Homo sapiens*, *Pan troglodytes*, *Sus scrofa*, Delphinapterus *leucas*, *Pteropus alecto*, *Mus musculus*, *Gallus gallus*, *Danio rerio*, *Esox lucius*, *Scleropages formosus, Saccharomyces cerevisiae*, *Geotrichum candidum, Tolypocladium paradoxum, Vigna unguiculate, Glycine soja, Arabidopsis thaliana, Zea mays, Klebsomidium nitens, Trachymymex cometzi* and *Drosophila melanogaster* were prepared with Clustal Omega using default parameters and visualized in JalView. The segment corresponding to the D1–D2 linker in Pex1 or Pex6 respectively, according to our homology model is boxed out and presented with 10 flanking residues to either side. The conservation score, ranging from 0 to 11, was calculated using the default method embedded into JalView. The columns corresponding to the discussed mutations localized in the D1-D2 linker are indicated by arrows.


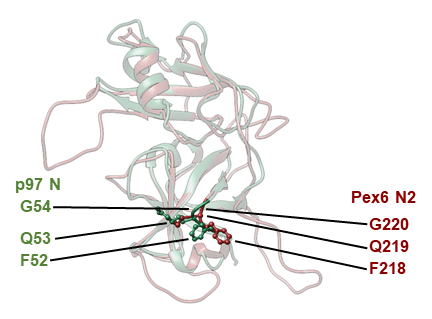


**Figure 3.** Superimposition of the Pex6 N2 domain and the p97 N domain (PDB: 5C18) highlighting residues 218 – 220 of Pex6 that structurally correspond to residues 52 – 54 of p97. Concerned amino acids are shown as sticks.
